# Global experience with PSMA-based alpha therapy in prostate cancer

**DOI:** 10.1007/s00259-021-05434-9

**Published:** 2021-06-26

**Authors:** Mike M. Sathekge, Frank Bruchertseifer, Mariza Vorster, Alfred Morgenstern, Ismaheel O. Lawal

**Affiliations:** 1 Department of Nuclear Medicine, University of Pretoria & Steve Biko Academic Hospital, Pretoria, South Africa; 2 Nuclear Medicine Research Infrastructure, Pretoria, South Africa; 3 European Commission, Joint Research Centre, Directorate for Nuclear Safety and Security, Karlsruhe, Germany

**Keywords:** Targeted alpha therapy, Prostate cancer, PSMA, Actinium-225, Bismuth-213

## Abstract

**Purpose:**

This review discusses the current state of prostate-specific membrane antigen (PSMA)-based alpha therapy of metastatic castration-resistant prostate cancer (mCRPC). With this in-depth discussion on the growing field of PSMA-based alpha therapy (PAT), we aimed to increase the interactions between basic scientists and physician–scientists in order to advance the field.

**Methods:**

To achieve this, we discuss the potential, current status, and opportunities for alpha therapy and strategies, attempted to date, and important questions that need to be addressed. The paper reviews important concepts, including whom to treat, how to treat, what to expect regarding treatment outcome, and toxicity, and areas requiring further investigations.

**Results:**

There is much excitement about the potential of this field. Much of the potential exists because these therapies utilize unique mechanisms of action, difficult to achieve with other conventional therapies.

**Conclusion:**

A better understanding of the strengths and limitations of PAT may help in creating an effective therapy for mCRPC and design a rational combinatorial approach to treatment by targeting different tumor pathways.

## Introduction

Prostate cancer is a biologically and clinically heterogeneous disease that is a leading cause of cancer death among men globally. Treatment for early-stage prostate cancer is radical prostatectomy or external beam radiotherapy. Prostate-specific antigen (PSA) levels rapidly fall to an undetectable level after treatment and can then be used as a tumor marker for disease surveillance [1]. More than half of these patients treated with curative radical prostatectomy or external beam radiotherapy will eventually have disease recurrence within a few years. Androgen blockade is a therapeutic option available in the treatment of recurrent prostate cancer. This is done to starve the prostate cancer cells of androgen, which it requires for growth. Effective androgen blockade halts disease progression but only temporarily as most patients become castration-resistant within a few years [2].

Therapy agents acting on different pathways, including taxane-based chemotherapy, next-generation anti-androgen, and radionuclide therapy with radium dichloride, have been found to prolong survival when applied in the treatment of mCRPC [2–4]. Despite the widespread utilization of these agents, mCRPC has remained a highly fatal disease. This, therefore, calls for continued effort in developing novel therapies to improve mCRPC-related mortality and morbidity. Prostate-specific membrane antigen (PSMA) is a membrane-expressed glycoprotein that is overexpressed on prostate cancer cells. The level of PSMA expression by prostate cancer cells is accentuated in metastatic and castration-resistant stages of the disease. This property of PSMA makes it an attractive target for imaging and therapy of the lethal forms of prostate cancer, metastatic, castration-resistant stage of the disease. PSMA-based radioligand therapy (PRLT) of mCRPC has been more commonly done using lutetium-177 (^177^Lu), a beta emitter complexed to PSMA ligand. The safety and efficacy of ^177^Lu-PSMA RLT for mCRPC treatment have been widely reported by many groups worldwide [5–7]. Most recently, the Thera-P trial, which is the first randomized phase 2 study of ^177^Lu-PSMA-617 compared with cabazitaxel in men with mCRPC, provides evidence that ^177^Lu-PSMA-617 is a potential alternative to cabazitaxel in men with mCRPC [8]. The trial results showed a greater PSA response rate and fewer toxicities in the ^177^Lu-PSMA-617 arm than the cabazitaxel arm [8].

Despite this remarkable response of mCRPC to ^177^Lu-PSMA-617, a significant proportion of patients may not respond to treatment [5–7]. Most patients who demonstrate a response to ^177^Lu-PSMA-617 initially may also experience disease progression. This has, therefore, led to an interest in the evaluation of the safety and efficacy of PSMA-based alpha therapy (PAT) as a therapeutic alternative for mCRPC patients who may be unsuitable for or resistant to ^177^Lu-PSMA-617.

## Prostate-specific membrane antigen (PSMA)

The evolution of knowledge on the biology of PSMA and its translation to therapy began with the development of the prostate cancer cell line LNCaP by Horoszewicz et al. in 1983 [9]. To date, several PSMA ligands have been developed for imaging and therapy of prostate cancer. The detailed discussion on these ligands is beyond the scope of this paper but has been recently presented by O’Keefe and colleagues [10]. PSMA, a type II 750-amino acid transmembrane protein (100–120 kDa), functions as a folate hydrolase I or glutamate carboxypeptidase II enzyme in the epithelial cells of the prostate gland [11–13]. Low-level diffuse physiologic PSMA expression occurs in the normal human prostate tissue, which is mainly localized to the cytoplasm and the apical side of the epithelial lining of the prostatic ducts but not basal epithelium, neuroendocrine, or stromal cells [14]. In dysplastic or frankly neoplastic prostate tissue, PSMA expression transfers from the apical membrane to the luminal surface of the ducts [15]. The main attraction for the use of PSMA for targeted therapy is its high level of expression that is increased by about 100- to 1000-folds higher in prostate cancer tissue compared with normal prostate tissue and the direct correlation between its level of expression and androgen independence, metastasis, and disease progression [16, 17]. As against what the name would suggest, PSMA expression is not specific for prostate cancer as it is overexpressed in the neovasculature of solid tumors but not in normal tissue vasculature. PSMA expression also occurs in several normal tissues, including the enterocytes of the small bowel, ductal cells of the proximal convoluted renal tubules, and salivary glands [18]. Following binding by PSMA radioligand, membrane-expressed PSMA undergoes clathrin-based internalization and, as such, can serve not only as an imaging biomarker but also as a target for radioligand therapy [6]. These characteristics make PSMA an appealing molecular target for theranostics of mCRPC [19].

## Alpha-emitting radionuclides for PSMA therapy

Alpha-emitting radionuclides have shown promising results as radiotherapeutic agents for the treatment of mCRPC. A detailed discussion on alpha emitters for radionuclide cancer therapy is discussed in detail in another article in this issue. Alpha particles for endo-radionuclide therapy have two distinct advantages over conventional therapies. Alpha particles are highly energetic and have a short-range in tissue (< 0.1 mm) corresponding to a few cell diameter. This combination ensures the deposition of a large amount of energy within a short radius leading to the effective killing of the targeted tumor with sparing of contiguous normal tissues. Also, the high linear energy transfer of alpha particles causes direct double-stranded deoxyribonucleic acid (DNA) damage and DNA cluster breaks that occur independent of the cell cycle phase or tissue oxygenation, which are also difficult to repair [20, 21]. Because of these attributes of alpha particles, therapy with alpha-emitting radionuclides has the potential to overcome resistance to PRLT with beta-emitting radionuclide or treatment with chemotherapeutic drugs [22, 23]. Despite the multitude of available alpha-emitting radionuclides, only a few of them have desirable characteristics that make them suitable for clinical application in targeted alpha therapy [24].

When applied in the treatment of mCRPC, the desired alpha-emitting radionuclide should target the entire spectrum of metastases, micrometastases, and overt metastatic disease in lymph nodes, the skeleton, and visceral organs. Accordingly, a couple of alpha-emitting radionuclides, including ^225^Ac, ^213^Bi, ^149^ Tb, ^212^Pb/^212^Bi, ^211^At, ^223^Ra, and ^227^Th, have been complexed to PSMA inhibitors and evaluated in preclinical and clinical studies for their efficacy and safety in the treatment of mCRPC (Table 1) [25]. Of the limited alpha-emitting radionuclides that are suitable for clinical application, ^225^Ac (physical half-life, T_1/2_ = 9.9d) and its short-lived daughter radionuclide, ^213^Bi (T_1/2_ = 46 min), have been most extensively studied [26] and will be discussed in detail. While the clinical translation of the preclinical work of the rest of the alpha-emitting PSMA targeting inhibitors appears promising based on the demonstrated antitumor activity, there are still some challenges associated with their use, as briefly highlighted below.

**Table 1:** Summary characteristics of alpha-emitting radionuclides suitable for targeted alpha therapy of prostate cancer

Radionuclide	Half-life	Emitted particles	Total α energy emitted per decay (MeV)	Range in tissue (μm)	LET (keV/μm)	Preclinical/clinical
^225^Ac	9.9 days	4α, 2β^−^	27.9	47–85	61–230	Preclinical/Clinical
^213^Bi	45.6 min	2α, 2β^−^	8.5	40–100	65–230	
^212^Pb/^212^Bi	10.6 h	1α, 2β^−^	7.9	40–100	61–230	Preclinical
^149^ Tb	4.1 h	1α,1ε/2ε, 1β^+^/2β^+^	0.7	25	140	
^227^Th	18.7 days	5α, 2β^−^	32.8	50–70	71–230	
^223^Ra	11.4 days	4α, 2β^−^	26.8	46–70	71–230	
^211^At	7.2 h	1α, 1ε	6.9	55–80	71–230	

^149^ Tb is a rare-earth element with a physical half-life of 4.1 h. It decays by emitting different particle, and non-particle radiations include α particles (3.97 MeV, 16.7%), electron capture (76.2%), positron emission (7.1%), gamma rays (165 keV, 26.4%), and X-rays. Therefore, it can be used for targeted alpha therapy (TAT), single-photon emission tomography (SPECT) imaging, and positron emission tomography (PET) imaging [25, 27]. The most striking limitation to the clinical application of this highly promising radionuclide relates to its limited available supply. The production and chemical separation of ^149^ Tb is fraught with many difficulties, a setback that may limit its clinical translation [28]. Long-lived daughter radionuclides in the decay scheme of ^149^ Tb complicate dosimetry and may contribute to radiation dose to patients. Alpha particle emission from ^149^ Tb is associated with high recoil energy that is sufficient to cause bond breakage leading to the systemic dissemination of daughter radionuclides and consequently whole-body radiation in general and bone marrow damage from the long-term accumulation of these free daughter radionuclides in the functional bone marrow [29].

^212^Pb decays by beta emission (physical half-life = 10.6 h) via its short-lived daughter radionuclides, ^212^Bi (T_1/2_ = 60.6 min) and ^212^Po (T_1/2_ = 0.3 µs) [25]. Two major limitations with the clinical application of ^212^Pb for targeted radioligand therapy relate to the high initial kidney uptake and consequently imparting a high renal dose as well as the high recoil energy, which is high enough to cause to up to 36% of ^212^Bi, one of its daughter radionuclides, to dissociate from the complexes [25, 30, 31].

^211^At has a physical half-life of 7.2 h. It decays by electron capture (58.3%) to ^211^Po (T_1/2_ = 0.52 s). Moreover, ^211^Po emits K X-rays that allow for the quantification of ^211^At radioactivity and scintigraphic imaging of ^211^At in vivo [32]. The challenge with ^211^At-PSMA is the high uptake in renal proximal tubules and late nephrotoxicity, as well as its limited availability [32, 33].

A detailed discussion of the radiochemistry and clinical evidence derived from ^223^Ra is presented in another article in this issue. Briefly, ^223^Ra is an alkaline earth metal, which forms very weak complexes [34]. To overcome this limitation, one study investigates the encapsulation of ^223^Ra into functionalized nanozeolites for TAT, with no definite results yet [35].

^227^Th has a physical half-life of 18.7 days and decays through radioactive ^223^Ra, which subsequently decays to stable ^207^Pb. During this decay scheme, five alpha particles are emitted [36]. Although ^227^Th-PSMA has been shown to have antitumor effect, reports on its impact on survival and its long-term toxicity are being awaited.

## Recoil effect

Alpha emission is a highly energetic process. Following the emission of an alpha particle, the resultant daughter radionuclide experiences recoil energy like the recoil effect felt when firing a gun. The energy of the recoil effect may be significant enough to cause the breakage of the bond between the daughter radionuclide and the ligand. When this happens, the released daughter radionuclide may be retained within the tumor and contribute to the overall cytotoxicity if the radioligand was internalized before bond breakage or circulate freely and be transported to other organs where it can accumulate and cause off-target damage of healthy organs [37]. The recoil effect in TAT may be mitigated by ensuring a fast tumor uptake of the radioligand and rapid renal excretion of unbound radioligand, intra-tumoral injection of the radioligand, or by encapsulation in a nano-carrier.

**Fig. 1:**
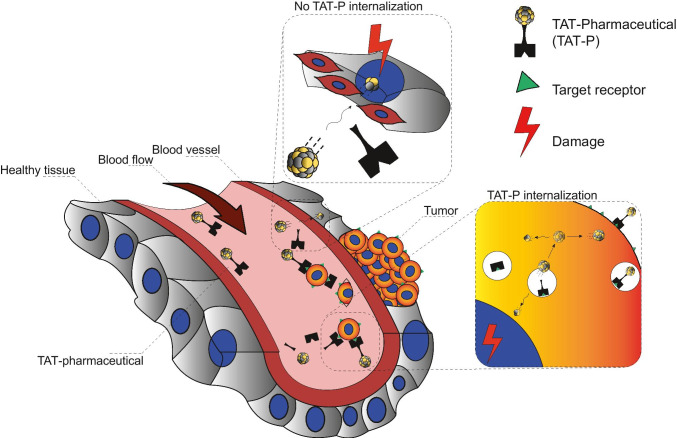
Schematic representation of two hypothetical scenarios describing the fate of the recoiling daughter radionuclide that gets released from the chelating moiety of TAT-P in vivo. The upper section labelled “No TAT-P internalization” depicts a daughter radionuclide that is released into the blood stream while causing either unspecific local damage to healthy tissue or travels further with the blood stream and causes analogical damage distantly elsewhere. The lower section labelled “TAT-P internalization” depicts TAT-P that specifically internalizes into the targeted tumor cell. The daughter radionuclide is then released with a high probability inside the tumor cell or to a minor extent might escape the tumor cell and cause damage not only to the target tumor cell but, depending on the traveled distance, to other cells as well

The rapid tumor uptake approach of the radiopharmaceutical has been used for targeting agents like PSMA as it is suitable for this strategy [38]. An excellent example of this strategy for PSMA-based TAT is the use of ^225^Ac as an atomic in vivo nanogenerator, in which ^225^Ac (T_1/2_ = 9.9 days) is administered for therapy leading to in vivo decay to ^213^Bi, its daughter radionuclide. This approach has overcome the limitation of the short physical half-life of ^213^Bi (T_1/2_ = 46 min). The higher number of alpha particles when the parent radionuclide is used for therapy (e.g., net α-energy of 28 MeV for ^225^Ac) compared with the daughter radionuclide ensures more significant tumoral cytotoxicity, constituting another attraction for the use of in vivo nanogenerators [26, 39]. Figure 1 from the paper by Roscher and colleagues shows the nuclear recoil effect during α-decay within radioligand for TAT [37].

## Current clinical experience with targeted alpha therapy of prostate cancer

### ^*213*^*Bi-PSMA*

^213^Bi (T_1/2_ = 46 min) is a mixed alpha/beta emitter that decays via beta emission to ^213^Po, an ultra-short-lived pure alpha emitter (T_1/2_ = 4.2 µs, E_α_ = 8.375 MeV, branching ratio = 97.8%). Alternatively, ^213^Bi decays to ^209^Tl via alpha particle emission (E_α_ = 5.549 MeV, 0.16%, E_α_ = 5.869 MeV, 2.01%, branching ratio = 2.2%). Both ^213^Po and ^209^Tl decay to ^209^Pb (a beta emitter with T_1/2_ of 3.25 h), which eventually decays to ^209^Bi (T_1/2_ = 1.9 × 10^19^ year). The alpha particle emitted by ^213^Po (with a path length in the tissue of 85 µm) [26] is the main driver of the cytotoxicity caused by ^213^Bi-based TAT as it contributes about 98% of the total energy due to all alpha particle emissions by ^213^Bi. The particle energy contributed by beta emission in the decay scheme of ^213^Bi is minimal (7.3%) [40]. The gamma photon emitted during ^213^Bi decay (440 keV, emission probability of 26.1%) is useful for SPECT imaging for biodistribution and dosimetric studies [26].

In the preclinical assessment of the efficacy of ^213^Bi-labelled PSMA inhibitor, effective cytotoxicity in cell lines overexpressing PSMA was demonstrated with ^213^Bi-J591 [41]. The radioimmunoconjugate was also found capable of determining effective cytotoxicity in cell lines overexpressing PSMA and in animal models of prostate cancer [41, 42]. No clinical studies demonstrating the efficacy and safety of ^213^Bi-J591 in the treatment of mCRPC have been reported yet.

Progress was made when Sathekge and colleagues presented the first-in-human treatment with two cycles and a cumulative activity of 592 MBq of ^213^Bi-PSMA-617 in a patient with mCRPC, with disease progression under conventional therapy [43]. TAT with ^213^Bi-PSMA-617 achieved a significant biochemical and imaging response [43]. Continued clinical application of ^213^Bi-PSMA-617 for the treatment of mCRPC has been limited. This has mainly been due to a preliminary dosimetric report, calculated in three patients with mCRPC submitted to PET/CT with ^68^ Ga-PSMA and extrapolated to the half-life of ^213^Bi, providing a therapeutic index for ^213^Bi-PSMA-617 that is inferior compared to ^225^Ac-PSMA-617 [44].

### ^*225*^*Ac-PSMA*

^225^Ac (T_1/2_ = 9.9 days) decays via a cascade of six short-lived daughter radionuclides to near-stable ^209^Bi (T_1/2_ = 1.9 × 10^19^ year) [45, 46]. The predominant decay path of ^225^Ac yields a net of 4 alpha particles (E_α_ of 5.8 MeV to 8.4 MeV, tissue range of 47 to 85 µm) and two beta particles (energy ranges of 0.6 to 1.6 MeV) [40]. Two gamma photons emitted during ^225^Ac decay (^221^Fr disintegration, 218 keV, 11.6% emission probability, and ^213^Bi disintegration, 440 keV, 26.1% emission probability) may provide a limited opportunity for imaging the in vivo distribution of the radionuclide [47–49].

## The practice of ^225^Ac-PSMA radioligand therapy

Much of the clinical practice of ^225^Ac-PSMA TAT is derived from lessons learned from the clinical application of ^177^Lu-PSMA for PSMA-based radioligand therapy (PRLT) of mCRPC. Practice guidelines on the application of ^177^Lu-PSMA-617 for PRLT of mCRPC have been published by many professional societies and groups [50–55].

### *Patient selection for *^*225*^*Ac-PSMA therapy*

The typical patient selected to undergo TAT with ^225^Ac-PSMA will be a patient with histologically confirmed prostate cancer whose disease has become castration-resistant and has progressed on conventional therapies. The decision to treat a patient with ^225^Ac-PSMA should be made in a multidisciplinary setting where the disease history, prior therapies, patient’s comorbid conditions and health state, available alternative treatments, and the patient’s wishes are thoroughly discussed. After this discussion, the multidisciplinary team must agree that ^225^Ac-PSMA therapy is the most suitable therapy option for the patient. Based on the known toxicity of ^225^Ac-PSMA therapy, sufficient vital organ reserve, especially of the bone marrow and kidneys, must be present before submitting a patient to TAT with ^225^Ac-PSMA. Acceptable organ reserve commonly applied in routine clinical practice is as follows:

- Bone marrow function: hemoglobin level > 8 g/dL; platelet count > 75 × 10^9^/L, white cell count > 3 × 10^9^/L
- Renal function: serum creatinine < 2 times the upper limit of normal

Prostate cancer becomes highly heterogeneous in the advanced stage of the disease, especially after multiple lines of therapy of mCRPC. Successful application of PSMA-based radioligand therapy, with alpha- or beta-emitting radionuclide, is based on sufficient expression of PSMA in all lesions. Therefore, a baseline imaging to demonstrate sufficient PSMA expression in tumor foci is vital in selecting patients for TAT with ^225^Ac-PSMA. A sufficient PSMA expression has traditionally been described as uptake above the physiologic PSMA uptake in the normal liver tissue. The level of PSMA expression is a significant predictor of response to ^225^Ac-PSMA therapy [56]. It is important to note that certain PSMA ligands such as PSMA-1007 have high hepatic background activity compared with most other PSMA ligands [57]. In the situation where ^18^F-PSMA-1007 PET/CT is obtained for baseline imaging, caution must be exercised in using hepatic background activity as reference standard to define sufficient PSMA expression in mCRPC lesions. Mediastinal blood-pool activity may be used as alternative internal reference in this situation. Given the known heterogeneity of PSMA expression within and between lesions [56], a determination must be made of the sufficient PSMA expression in all lesions. This determination becomes more critical in TAT considering the shorter tissue range of alpha particles which may fail to induce a significant crossfire effect in a tumor lesion with a widely dispersed pattern of PSMA expression.

Except in the circumstances where there is a failure of response to ^177^Lu-PSMA therapy, or mCRPC has progressed after an initial response to ^177^Lu-PSMA, in most instances, patients who qualify to receive ^225^Ac-PSMA may be found suitable for ^177^Lu-PSMA as well. It, therefore, becomes pertinent to design a rational approach to determine the more suitable of the two therapy options for each patient. The red marrow is a common site of prostate cancer metastases. It is not unusual to encounter diffuse metastases of prostate cancer to the axial skeleton in a manner typical of superscan [58]. In such instances, it may be prudent to treat such patients with ^225^Ac-PSMA due to its shorter tissue range to preserve the functional marrow [59]. Baseline PSMA imaging with either SPECT or PET technique helps make this selection [57, 60, 61].

Another factor that may be of paramount consideration in selecting between alpha- and beta-emitting radioligand for targeted therapy of mCRPC is the size of lesions. The dose delivered to a tumor mass by radionuclide therapy is directly proportional to the size [62]. Due to the limited radiation dose delivered to tissue per micron of tissue traversed by beta particles, ^177^Lu-PSMA is, therefore, unlikely to be effective in eliminating small lesions due to mCRPC [63]. These patients with suspected or confirmed sub-centimeter lesions or micrometastases of mCRPC may be more effectively treated with ^225^Ac-PSMA than ^177^Lu-PSMA.

Recent prospective trials of ^177^Lu-PSMA-617 in mCRPC have shown the value of adding ^18^F-fluorodeoxyglucose (^18^F-FDG) PET/CT to the initial assessment of patients being worked up for PRLT [7, 8]. In one of those trials, 16% of patients failed eligibility due to discordance between ^18^F-FDG PET and ^68^ Ga-PSMA PET; metastatic lesions visualized on ^18^F-FDG PET were not PSMA-avid on ^68^ Ga-PSMA PET [7]. Superselection of patients for PRLT in this manner appears to result in a better PSA response rate and should be performed if available resources are permitting [7, 8, 55]. No published evidence is available currently on the impact of the addition of ^18^F-FDG PET/CT in the assessment of patient suitability for ^225^Ac-PSMA. In our practice and in most other centers administering ^225^Ac-PSMA for therapy of mCRPC across the world, ^18^F-FDG PET is not routinely used in the selection of patients for TAT.

### Preparation for therapy administration

^225^Ac-PSMA should be administered following national regulations on the safe use of unsealed radiation sources in a facility that is suitably equipped and staff trained in administering radionuclide therapy and managing accidental radiation contamination of persons and site. In preparation for therapy administration, baseline blood tests should be obtained for efficacy and toxicity monitoring. These baseline blood tests should include the following:

- Serum PSA.
- Full blood count.
- Serum electrolyte, urea, and creatinine.
- Estimated glomerular filtration rate (eGFR).
- Liver function tests.
- Dynamic renal scintigraphy is indicated to demonstrate dilatation or obstruction in the renal collecting system. Procedures to relieve obstruction are indicated in patients with obstruction in their renal collecting system to prevent an undue increase in renal dose.

Consenting forms an essential aspect of patient preparation for radionuclide therapy. Consenting includes but is not limited to providing comprehensive information regarding ^225^Ac-PSMA in terms of efficacy and side effects, the goal of treatment, and alternative therapy options available to the patient. It must be made clear to the patients that ^225^Ac-PSMA is not yet an approved agent for therapy of mCRPC and is applied on a compassionate ground or as part of a clinical trial in qualifying patients.

To prevent additive toxicity on the functional bone marrow, all myelotoxic therapies must be discontinued for a minimum of 6 weeks, and hematologic indices return to an acceptable threshold as indicated above before ^225^Ac-PSMA administration. No contraindication exists to co-administration of androgen deprivation therapy (ADT) with ^225^Ac-PSMA. Continued use of ADT should be based on clinical indication. There is no indication to discontinue bone-supporting agents such as bisphosphonates and RANK ligand inhibitor (denosumab).

### Therapy administration

^225^Ac-PSMA is administered intravenously via a free-hand slow bolus injection given over 20 to 30 s. To enhance renal excretion of unbound radioligand, 1 to 2 l of an intravenous physiologic solution such as Ringer’s lactate or normal saline should be administered for 4 h, commencing 30 min before ^225^Ac-PSMA administration. This rate and volume of fluid administration must be tailored towards the prevailing medication conditions in the patient. Certain conditions such as congestive cardiac failure, when present, may preclude the generous hydration of the patient. Urethral catheterization for 48 h may be indicated in the incontinent patient to prevent radiation contamination of self and environment.

^225^Ac-PSMA presents no significant radiation burden to people in close contact with the treated patients due to the low activity of the radioligand administered for treatment (one-thousandth times lower than the activity of ^177^Lu-PSMA). Therefore, the decision to treat a patient with ^225^Ac-PSMA on either an in- or outpatient basis should be in accordance with the national laws. In climes where the national laws are permitting, ^225^Ac-PSMA therapy can be administered on an outpatient basis.

### Follow-up and response assessment

Follow-up visits must be scheduled to monitor toxicities and determine treatment efficacy. Baseline blood tests (serum PSA; serum urea, electrolytes and creatinine levels, creatinine clearance for eGFR, liver function tests) should be performed as required during follow-up for safety and efficacy assessments. Treatment is repeated after 8 weeks. Follow-up blood tests should be done within 2 weeks of the next treatment cycle. Treatment is repeated for up to 6 cycles or more provided there is continued demonstrable efficacy in the absence of severe toxicity.

The assessment of treatment efficacy should be done in three domains: PSA response, radiological response, and clinical response [64]. PSA response is determined as recommended by the Prostate Cancer Clinical Trial Group as follows [65]:

- PSA response: PSA decline ≥ 50% from baseline measured twice 3 to 4 weeks apart
- PSA progression: rise in PSA by 25% from the nadir and an increase of at least 2 ng/mL
- Stable PSA: decline < 50% or rise < 25%

Radiological response is traditionally determined using morphologic imaging with CT or magnetic resonance imaging (MRI) using the Response Evaluation Criteria in Solid Tumors 1.1 (RECIST 1.1). Some recent studies have shown the potential of ^68^ Ga-PSMA PET/CT for response assessment in patients treated for mCRPC [66–68]. ^68^ Ga-PSMA criteria for response assessment in men treated for prostate cancer have recently been published by a multidisciplinary team of urologists, radiologists, and nuclear medicine physicians [69]. ^68^ Ga-PSMA PET/CT may find greater application in response assessment of prostate cancer therapy as it has already been shown to outperform conventional imaging with CT and bone scan in localizing prostate cancer lesions [70, 71]. It must be borne in mind, however, that one of the mechanisms by which mCRPC become resistant to PSMA-based radioligand therapy with alpha- or beta-emitting radionuclides is by downregulating PSMA expression [72, 73]. Reliance on imaging findings on ^68^ Ga-PSMA PET imaging alone may lead to failure to confirm disease progression when lesions downregulate their expression of PSMA.

Clinical response is based on improvement in disease-related symptoms. Clinical assessment should also be done to determine treatment-related toxicities. The clinical impact of prostate cancer therapy is best assessed objectively using validated questionnaires such as the European Organization for Research and Treatment of Cancer Quality of Life Questionnaire (EORTC-QLQ), Patient-Reported Outcomes Measurement Information System, Brief Pain Inventory, etc.

## Current evidence for the efficacy of ^225^Ac-PSMA therapy in mCRPC

The first set of studies demonstrating the efficacy of ^225^Ac-PSMA in the treatment of mCRPC came from Heidelberg, Germany. A report of two patients who had exhausted available conventional therapies, one of whom was deemed ineligible for ^177^Lu-PSMA due to diffuse red marrow metastases of mCRPC and the other experienced disease progression on ^177^Lu-PSMA therapy, was the first to show the efficacy of ^225^Ac-PSMA-617 in large volume metastases of mCRPC [38]. In the patient with diffuse marrow metastases of mCRPC, normalization of serum PSA and ^68^ Ga-PSMA PET/CT imaging findings occurred after four cycles of ^225^Ac-PSMA-617 with no significant change in hematologic indices. In the other patient with radioresistant to beta-emitting radionuclide therapy with ^177^Lu-PSMA therapy, a large volume of peritoneal metastases invading into the liver resolved, and serum PSA dropped to below detectable limit after three cycles of ^225^Ac-PSMA-617 [38]. This report provided the first preliminary insights into the capability of ^225^Ac-PSMA TAT for mCRPC, including its safety and efficacy in the setting of diffuse red marrow metastases, its effectiveness in the setting of radioresistant to PRLT with a beta-emitting radionuclide, its ability to eradicate large volume metastases, and its efficacy as a last-line therapy in patients who have failed multiple lines of therapy for mCRPC.

In 2017, in collaboration with scientists at the Joint Research Centre of the European Commission, the Heidelberg group published a follow-up dose-escalation study to define the optimum activity required to achieve the maximum antitumor effect and the dose-limiting organs [74]. A group of 14 patients was treated with an escalating activity of ^225^Ac-PSMA-617, from 50 KBq/Kg body weight to 200 KBq/Kg. Xerostomia was the commonest treatment-related side effects prevalent in patients treated with 100KBq/Kg and above. Based on their findings, the authors arrived at 100 KBq/Kg as the maximum tolerable activity for ^225^Ac-PSMA-617 and xerostomia as the dose-limiting toxicity [74]. This study has influenced the global practice of TAT of mCRPC as findings from it have been used to guide empirical dosing of ^225^Ac-PSMA.

The sequencing of agents used in the treatment of mCRPC is critical as agents applied earlier in the disease course achieve better response compared with agents applied later in the treatment sequence. The most recent and the largest study from the Heidelberg group and their collaborators demonstrated superior efficacy of ^225^Ac-PSMA-617 applied as a last-line therapy agent in heavily pretreated patients compared with approved agents that were applied earlier in the treatment sequence of the patients [75]. Using a swimmer-plot analysis, the group showed the relative durations of tumor control induced by different life-prolonging therapies of mCRPC. The average duration of tumor control induced by any first-, second-, third-, or fourth-line agent, regardless of the agent, was 8.0, 7.0, 6.0, and 4.0, respectively. The average duration of tumor control induced by ^223^RaCl_2_, cabazitaxel, enzalutamide, docetaxel, and abiraterone, regardless of the time the particular agent was applied in the treatment sequence, was 4.0, 6.0, 6.5, 6.5, and 10.0 months, respectively. ^225^Ac-PSMA-617 induced an average duration of tumor control of 9.0 months in a cohort of patients 85, 70, 60, 22.5, and 12.5% of whom had prior therapy with abiraterone, docetaxel, enzalutamide, ^223^RaCl_2_, and cabazitaxel, respectively [75]. This study added important insights to the knowledge on ^225^Ac-PSMA therapy in mCRPC, confirming its antitumor activity in patients who have exhausted or have limited therapy options available for their treatment. In addition, the study showed, perhaps, a better duration of disease control inducible by ^225^Ac-PSMA therapy compared with the available approved agents with life-prolonging capability.

**Fig. 2:**
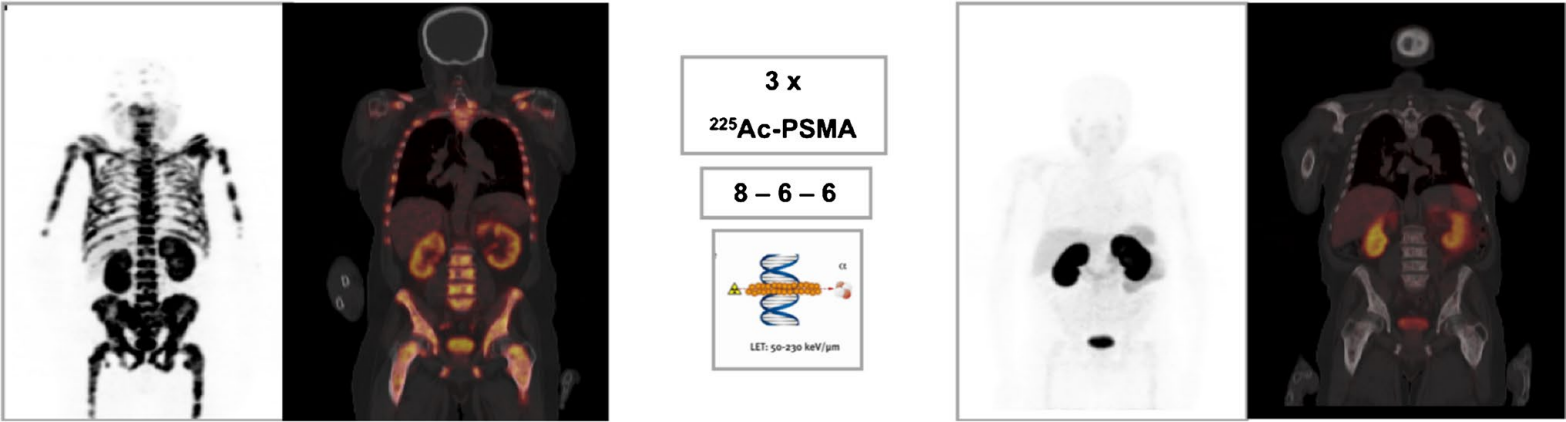
A 67-year-old-male with extensive skeletal metastases, Gleason score = 4 + 5. He was treated with 225Ac-PSMA-617 following disease progression on conventional therapy. The bone marrow remained stable after treatment

The largest series so far published on the application of ^225^Ac-PSMA-617 for mCRPC was from Pretoria, South Africa [76]. In a cohort of 73 men with mCRPC who were treated with a total of 210 cycles of ^225^Ac-PSMA-617 (median treatment cycle = 3, range = 1–8), PSA response (decline in serum PSA of 50% or more) was seen in 70% of patients, while any decline in serum PSA was seen in 82% of the patients. There was complete normalization of imaging findings assessed by ^68^ Ga-PSMA PET/CT in 28.8% of the patients. The progression-free survival (PFS) and overall survival (OS) were 15.2 (95% CI, 13.1–17.4) months and 18 (95% CI, 16.2–19.9) months, respectively [76]. This study showed, in a relatively large cohort of patients, the efficacy, durability of disease control, and survival inducible by ^225^Ac-PSMA-617 therapy of mCRPC (Fig. 2).

The Indian experience with the use of ^225^Ac-PSMA-617 in mCRPC has recently been reported from New Delhi [77]. In a cohort of 28 patients, 89% of patients had any PSA decline 8 weeks after the first cycle of ^225^Ac-PSMA-617. After administering a total of 85 cycles of ^225^Ac-PSMA-617 in the 28 patients, 78.6% of the patients achieved any PSA decline, while 39% of patients achieved a PSA decline of 50% or more. Despite this relatively low PSA response rate, a median PFS and OS of 12 (95% CI, 9–13) months and 17 (16, not reached) months were attained. Clinical response assessed by visual analog score, analgesic score, Eastern Cooperative Oncology Group performance status, and Karnofsky performance status showed significant improvement in favor of clinical benefit of ^225^Ac-PSMA-617 for mCRPC treatment [77]. This Indian study advanced knowledge by providing evidence to support the clinical benefits of ^225^Ac-PSMA-617 in mCRPC as it relates to improvement in the severity of pain, a corresponding reduction in the need for analgesic, and an improvement in patients’ performance of the activities of daily living.

A second Indian study from Chandigarh has also shown the impact of ^225^Ac-PSMA-617 on the health-related quality of life of 11 patients who had failed treatment with two or more lines of approved agents for mCRPC [78]. After a median of 2 cycles of ^225^Ac-PSMA-617, 46% of patients achieved a PSA decline of 50% or more. Health-related quality of life assessed by a validated questionnaire assessing physical wellbeing, emotional wellbeing, treatment-related side effects, and functional wellbeing showed an overall significantly improvement post-treatment with ^225^Ac-PSMA-617. Among the four domains evaluated by the questionnaire, significant improvement occurred in the physical and emotional wellbeing domains. The physical symptoms that showed improvement with ^225^Ac-PSMA-617 therapy were pain, difficulty with urination, fatigue, and restriction in physical activity [78]. The positive impact of ^225^Ac-PSMA-617 on patients’ quality of life was also demonstrated in a report from Nijmegen, the Netherlands, of Dutch patients treated at Heidelberg, Germany, with ^225^Ac-PSMA-617 for mCRPC [79]. The patients reported outcome assessed by the EORTC-QLQ-30 and BM-22 questionnaires showed a significant decrease in pain complain and a corresponding decrease in the need for analgesics. There was a progressively increase in the scores for physical and role functioning scales from after therapy completion [79]. Put together, these three studies show a consistent improvement in the quality of life of patients with mCRPC who were treated with ^225^Ac-PSMA-617.

In the last few years, different groups have reported insightful cases of treatment outcome of mCRPC with ^225^Ac-PSMA-617, including remarkable response in patients with visceral metastases [80, 81], long-lasting remission [82], and some rare treatment-related side effects [83, 84]. Table 2 summarizes clinical studies reporting the treatment outcome of mCRPC with ^225^Ac-PSMA TAT.

**Table 2:** Summary of clinical studies on PSMA-based targeted alpha therapy of prostate cancer

Radioligand	First author, year of publication	Number of patients	% of patients with PSA decline of ≥ 50% (n/N)	Comments
^213^Bi- PSMA-617	Sathekge et al., 2017 [43]	1	100% (1/1)	Remarkable response of mCRPC to 2 cycles of ^223^Bi-PSMA-617
	Kratochwil et al., 2016 [38]	2	100% (2/2)	First evidence of safety and efficacy of ^225^Ac-PSMA-617 therapy in mCRPC
	Kratochwil et al., 2017 [74]	14	44% (4/9)	Dose-escalation study establishing salivary gland as the dose-limiting organ and 100 KBq as the maximum tolerable activity for ^225^Ac-PSMA therapy
	Kratochwil et al., 2018 [75]	40	63% (24/38)	^225^Ac-PSMA-617 show longer duration of disease control than approved agents applied earlier in the treatment sequence of mCRPC
	Sathekge et al., 2019 [79]	1	100% (1/1)	Complete resolution of cerebral and skeletal metastases of mCRPC following ^225^Ac-PSMA-617 therapy
	De Medeiros et al., 2019 [83]	1	100% (1/1)	A patient with large volume metastases of mCRPC developed tumor lysis syndrome following treatment with ^225^Ac-PSMA-617
^225^Ac-PSMA-617	Sathekge et al., 2019 [92]	17	88% (15/17)	Remarkable and durable response of mCRPC to ^225^Ac-PSMA-617 in chemotherapy-naïve men
	Sathekge et al., 2020 [76]	73	70% (51/73)	Largest series on the efficacy and safety of ^225^Ac-PSMA-617 in mCRPC. Shows prior ^177^Lu-PSMA therapy as a poor predictor of survival
	Yadav et al., 2020 [77]	28	39% (11/28)	Therapy of mCRPC with ^225^Ac-PSMA-617 was associated with an improvement in global health status of treated patients
	Feuerecker et al., 2020 [88]	26	65% (17/26)	Remarkable PSA response in heavily pretreated patients who had failed ^177^Lu-PSMA therapy Duration of PSA response was short No significant change in the global health status of the patients following treatment
	Khreish et al., 2020 [89]	20	65% (13/20)	^225^Ac-PSMA-617/^177^Lu-PSMA-617 applied in tandem induced remarkable PSA response in patients with poor response to prior ^177^Lu-PSMA-617 monotherapy
	Satapathy et al., 2020 [78]	11	46% (5/11)	The improvement in patients’ quality of life following ^225^Ac-PSMA-617 therapy occurs mostly in the physical (improvement in pain, difficulty with micturition, fatigue, and physical activity) and emotional domains
	Van der Doelen et al., 2020 [79]	13	69% (9/13)	Also confirms improvement in quality of life following ^225^Ac-PSMA-617 therapy
	Rathke et al., 2020 [82]	1	100% (1/1)	Patient with history of mCRPC remained in remission 5 years after therapy with ^225^Ac-PSMA-617
	Rosar et al., 2021 [90]	17	29%	Antitumor activity of a single course of ^225^Ac-PSMA-617/^177^Lu-PSMA-617 administered in tandem to patients whose disease progressed after ^177^Lu-PSMA-617 monotherapy Good concordance in response assessed by serum PSA and molecular metrics derived from ^68^ Ga-PSMA PET/CT
	Pelletier et al., 2021[84]	2	NA	Two patients developed progressive renal failure following treatment with ^225^Ac-PSMA-617 therapy
	Maserumule et al., 2021 [81]	1	100% (1/1)	Complete remission of extensive bilateral pulmonary metastases of mCRPC induced by ^225^Ac-PSMA-617 therapy
^225^Ac-PSMA-I&T	Ilhan et al., 2020 [86]	1	100% (1/1)	A patient with mCRPC, treated with 10 cycles of ^177^Lu-PSMA-617. He developed resistance to ^177^Lu-PSMA-617 and was treated with 2 cycles of ^225^Ac-PSMA-I&T, to which he responded
	Zacherl et al., 2020 [87]	14	50% (7/14)	First series reporting the efficacy of ^225^Ac-PSMA-617 in men with mCRPC

PSMA-617 is the most commonly applied PSMA inhibitor for PRLT of prostate cancer. PSMA-I&T is a less commonly used PSMA inhibitor with similar biokinetics and excellent binding capacity at a nanomolar concentration as PSMA-617 [11, 85]. Ilhan and colleagues from Munich, Germany, reported a case showing a good antitumor effect of ^225^Ac-PSMA-I&T in a patient previously treated with 10 cycles of ^177^Lu-PSMA-617 [86]. The group subsequently published their experience on the use of ^225^Ac-PSMA-I&T in 14 men with mCRPC. After a total of 34 cycles of ^225^Ac-PSMA-I&T (median = 7, range = 1–5), 78.6% and 50% of patients had any PSA decline and ≥ 50% PSA decline, respectively [87].

## Efficacy of ^225^Ac-PSMA in the post^−177^Lu-PSMA therapy setting

^177^Lu-PSMA is the more commonly applied agent for radionuclide therapy of mCRPC due to its wider availability and a more robust clinical experience with its use. ^177^LuPSMA is an effective treatment modality for mCRPC. However, a significant proportion of patients will not respond, and of those who respond, many will experience disease progression after several months [5–7]. ^225^Ac-PSMA TAT has been applied as a salvage therapy in patients who do not respond to and experience disease progression after an initial response to ^177^Lu-PSMA therapy on the background that ^225^Ac-PSMA can overcome radioresistant to ^177^Lu-PSMA in mCRPC [38]. Many of the published series on ^225^Ac-PSMA therapy of mCRPC have included patients with prior history of ^177^Lu-PSMA therapy. In the Pretoria series by Sathekge et al. [76], patients with a prior history of ^177^Lu-PSMA therapy had a significantly shorter PFS (5.1 months, 95% CI, 3.8–6.5 months versus 16.5 months, 95% CI, 14.3–18.7 months) compared with ^177^Lu-PSMA therapy-naïve patients. On multivariate analysis, prior ^177^Lu-PSMA therapy was significantly associated with shorter PFS [76]. In the Indian series by Yadav and colleagues, PFS and OS were shorter in patients with a history of prior ^177^Lu-PSMA therapy compared with patients without; the difference did not reach statistical significance, however, probably due to the small study population [77].

To evaluate the efficacy of ^225^Ac-PSMA in the post-^177^Lu-PSMA setting further, the authors from Munich, Germany, and their collaborators reported their experience with the use of ^225^Ac-PSMA-617 therapy in heavily pretreated patients who had failed a median of six prior regimens for mCRPC, including prior ^177^Lu-PSMA therapy [88]. Any PSA decline and PSA decline of 50% or more were seen in 88.5% (95% CI, 70–97%) and 65.4% (95% CI, 46–81%), respectively. The median PSA-PFS and OS were 3.5 (95% CI, 1.8–11.2) months and 7.7 (95% CI, 4.5–12.1) months. The presence of liver metastases at the initiation of treatment was a significant predictor of a shorter PSA-PFS and OS. Treatment of mCRPC with ^225^Ac-PSMA in this cohort of patients that had progressed on ^177^Lu-PSMA therapy did not produce any measurable changes on the quality of life of the patients assessed by the EORTC-QLQ30 questionnaire [88]. The results from this study clearly show a good PSA response to ^225^Ac-PSMA in the post-^177^Lu-PSMA setting, albeit for a short duration without a corresponding improvement in the global health status of the patients. Randomized controlled trials will be needed in the future to stratify patients to either ^177^Lu-PSMA or ^225^Ac-PSMA so that the better therapy is administered in the treatment sequence when it is likely to have the best impact.

Two interesting studies have been recently published on the efficacy of ^225^Ac-PSMA in patients with prior history of ^177^Lu-PSMA therapy. Rather than applying ^225^Ac-PSMA alone, ^177^Lu-PSMA and ^225^Ac-PSMA were applied tandem for mCRPC in the two studies. In the study by Khreish et al. from Homburg, Germany, 20 patients who had demonstrated insufficient response to ^177^Lu-PSMA monotherapy were treated with one course of ^225^Ac-PSMA-617/^177^Lu-PSMA-617 applied in tandem (mostly on consecutive days) [89]. PSA response was achieved in 50% of patients 6 to 8 weeks after tandem therapy. Response to tandem therapy was not significantly different between patients who showed an earlier response to prior ^177^Lu-PSMA-617 therapy before developing resistance (n = 12) versus those patients who never responded to the prior ^177^Lu-PSMA-617 therapy (n = 8) [89]. This study highlighted the antitumor activity of ^225^Ac-PSMA-617 against two patterns of radioresistant to beta-emitting radionuclide therapy of mCRPC. The Homburg group has also reported their experience with one course of ^225^Ac-PSMA-617/^177^Lu-PSMA-617 administered in tandem to a different cohort of patients with prior history of response to ^177^Lu-PSMA-617 therapy [90]. In this latter study, response was assessed by serum PSA and functional parameters derived from ^68^ Ga-PSMA PET/CT. PSA response and partial response assesses by ^68^ Ga-PSMA PET/CT was seen in 29.4% of patients, with a 70.6% concordance in response assessed by serum PSA and ^68^ Ga-PSMA PET/CT [90]. This latter study, in addition to showing the antitumor activity of tandem ^225^Ac-PSMA-617/^177^Lu-PSMA-617 in patients with disease progression after an initial response to ^177^Lu-PSMA-617 monotherapy, demonstrated the potential role of molecular indices derived from ^68^ Ga-PSMA PET/CT for response assessment and their concordance with response assessment with serum PSA.

## ^225^Ac-PSMA: upfront application in the chemotherapy-naïve setting

Despite the availability of multiple life-prolonging therapy options for mCRPC, there is no consensus on the sequence at which to apply them for treatment. It is known that agents applied earlier in the treatment sequence achieve better responses than agents applied later in the sequence. Randomized control trials are needed to determine the rightful place of each agent in the treatment sequence of mCRPC. A couple of studies have shown a better response of PRLT with ^177^Lu-PSMA in the chemotherapy-naïve patients [6, 91].

**Fig. 3:**
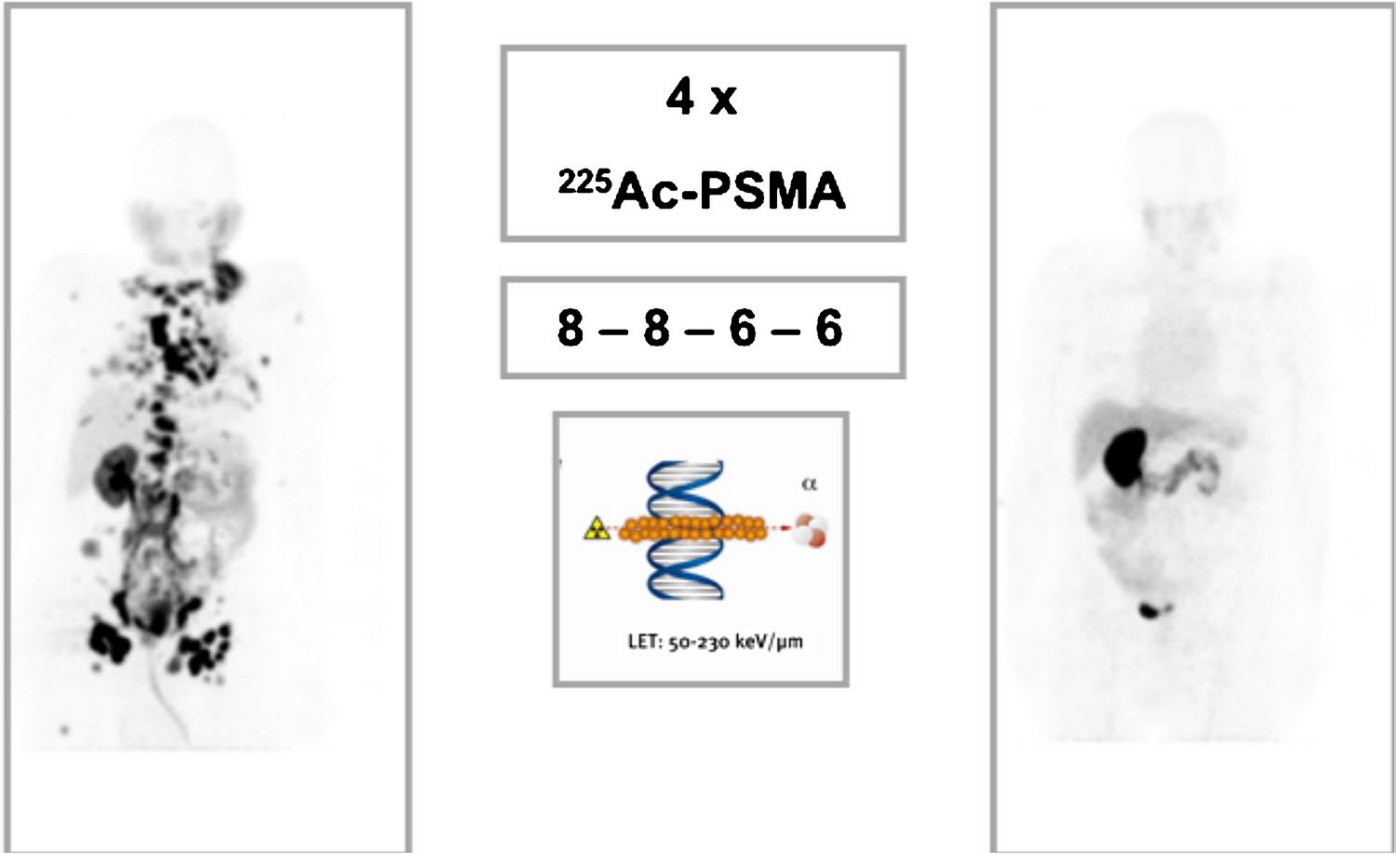
A 69-year-old-male with extensive skeletal and soft tissue metastases of metastatic castration-resistant prostate cancer, Gleason score = 4 + 4. He was chemotherapy-naïve at the time of 225Ac-PSMA-617 therapy. He had a durable response to therapy

Our group in Pretoria, South Africa, published a unique cohort of chemotherapy-naïve men with mCRPC who had upfront treatment with ^225^Ac-PSMA [92]. These were men who either declined treatment with taxane-based chemotherapy agents or had no access to them. In 88% of patients, serum PSA declined by 50% or more after a median of three cycles of ^225^Ac-PSMA-617. In 41% of patients, serum PSA declined to below detectable limit and remained so after a median follow-up duration of 12 months. In 65% of the patients, there was normalization of ^68^ Ga-PSMA PET/CT findings with tracer uptake in all malignant lesions reducing to background activity level (Fig. 3). While this remarkable response is exciting and holds much promise for applying ^225^Ac-PSMA-617 in treating men with mCRPC, it may also represent a response achieved in less aggressive disease. mCRPC evolves, acquiring more aggressive behavior as different lines of treatments are applied to it.

## Toxicity

Treatment-related side effects of ^225^Ac-PSMA TAT can be direct or indirect. Direct toxicity results from damage induced by alpha particles in organs expressing PSMA or organs involved with the excretion of the radioligand. Organs expressing significant PSMA expression include salivary glands, lachrymal glands, enterocytes of the small bowel, and the epithelial lining of the proximal convoluted tubules [93, 94]. The salivary glands are the dose-limiting organs at the activities administered for ^225^Ac-PSMA TAT [60]. Salivary gland damage causing xerostomia is, therefore, the commonest treatment-related toxicity of ^225^Ac-PSMA TAT observed in up to about 72.7% of treated patients [95]. Xerostomia leads to many other indirect side effects of treatment, including dysgeusia, reduced food intake, dyspepsia, weight loss, and constipation [76].

Salivary gland uptake of PSMA radioligand occurs via specific receptor binding and non-specific trapping [96]. The severity of xerostomia and its impact on the quality of life of patients treated with ^225^Ac-PSMA has led to a lot of interest in designing ways to mitigate against its incidence and severity. None of the currently tested methods is universally effective in addressing xerostomia as a treatment-related side effect of ^255^Ac-PSMA TAT. The simplest intervention applied for reducing salivary gland toxicity is by external cooling of the gland during therapy administration. This intervention is premised on the theory of reduced radioligand delivery to the major salivary glands due to the vasoconstrictive effect induced by external cooling. The effectiveness of this intervention is still under debate [97, 98]. Competitive inhibition for binding at PSMA receptor expressed in normal organs has been attempted with cold PSMA or PSMA congeners such as monosodium glutamate as another way to reduce off-target PSMA radioligand uptake in the salivary glands [99–103]. While this competitive inhibition may successfully reduce off-target PSMA radioligand in normal organs such as the salivary gland, a reduction in tumor uptake is possible [102]. Ligand modification in which radioligand preferentially binds to tumor-expressed PSMA but not PSMA expressed in normal organs appears exciting and. if successful, may have a great impact on the practice of PRLT of mCRPC [104]. Other pharmacologic interventions that have been tried for their ability to reduce salivary gland uptake of PSMA radioligands are botulinum injection, anti-cholinergic use, and injection of local anesthetic agents [105, 106]. Sialendoscopy with dilatation, saline irrigation, and steroid injection into the ducts of major salivary glands has been attempted to ameliorate the impact of xerostomia in patients treated with ^225^Ac-PSMA TAT [107].

Treatment de-escalation is an intervention that has been specifically utilized in ^225^Ac-PSMA TAT to reduce the incidence and severity of treatment-induced xerostomia. In this strategy popularized by our group in Pretoria, treatment commences with 8 MBq of ^225^Ac-PSMA. ^68^ Ga-PSMA PET/CT is used to assess for the volume of residual disease and response to therapy. Administered activity is reduced to 6 or 4 MBq in subsequent treatment cycles according to the volume of residual tumor load. This strategy is based on the principle of tumor sink effect in which more radioligand is available for binding in normal organs with reducing tumor bulk induced by successful treatment [108]. This strategy has been successful as none of our patients has experienced grade III xerostomia or discontinued ^225^Ac-PSMA therapy due to dry mouth [76, 92]. A second specific approach to reduce the incidence and severity of ^225^Ac-PSMA-induced xerostomia is tandem administration of ^225^Ac-PSMA and ^177^Lu-PSMA. This approach popularized by the Homburg group uses a lower activity of ^225^Ac-PSMA (an average of 5.3 MBq) combined with a standard activity of ^177^Lu-PSMA to achieve an optimum antitumor effect without the undesirable severe xerostomia inducible by a standard activity of ^225^Ac-PSMA [89, 90]. In one of their studies reporting the outcome of ^225^Ac-PSMA-617/^177^Lu-PSMA-617 tandem therapy, 40% and 25% of patients reported grades I and II xerostomia, respectively [89]. This relatively lower frequency of very mild to mild xerostomia was seen despite a prior history of pretreatment with ^177^Lu-PSMA-617 in the cohorts (median = 4 cycles, range = 1–13 cycles) [89].

Despite being administered in heavily pretreated patients with limited bone marrow reserve and in patients with predominant diffuse red marrow metastases, ^225^Ac-PSMA TAT is rarely associated with grades III/IV hematologic toxicities [38, 75, 76]. A recent meta-analysis of 10 studies, including 256 patients treated with ^225^Ac-PSMA, ≥ grade3 anemia, leucopenia, and thrombocytopenia, was seen in 12.8%, 8.3%, and 6.3%, respectively [95]. The proportions of patients who developed any grade of hematologic toxicity following ^177^Lu-PSMA RLT are 4–85% for anemia, 3–53% for leucopenia, and 5–47% for thrombocytopenia [5]. The shorter path length of alpha particles compared with beta particles ensured that high energy is deposited within the targeted bone metastases with a limited dose delivered to the surrounding red marrow [109].

Renal toxicity is another potential side effect of ^225^Ac-PSMA therapy [84]. In the meta-analysis of 10 studies by Satapathy and colleagues, only 3.8% of patients were reported to have ≥ grade III renal toxicity [95].

## Resistance and mutation

Several genotypic and phenotypic characteristics have been noted to drive resistance to TAT with ^225^Ac-PSMA-617. The most used phenotypical characteristic to select patients for PRLT is the level of PSMA expression. Low PSMA expression corresponds to a high tumor proliferative index and poor survival after ^225^Ac-PSMA TAT [79]. Therapy-induced neuroendocrine differentiation is another histologically determined phenotype that portends poor treatment outcomes [79]. Visceral metastases occur at a very advanced stage of mCRPC. Metastases to soft tissue visceral, especially to the liver, are poor prognostic indicators [88].

Early evidence from genomic and proteomic studies has shown a prevalence of mutations in the DNA damage repair machinery in the tumor cells of prostate cancer resistant to TAT with ^225^Ac-PSMA. The p53 gene, acting via cyclin and cyclin-dependent kinases, functions as the guardian of the genome by halting cell cycle progression in response to DNA damage. In the preclinical study by Stuparu and colleagues, loss of TP53 was associated with poor response to TAT with ^225^Ac-PSMA-617 [110]. In the earlier clinical study by Kratochwil et al., patients with mCRPC resistant to ^225^Ac-PSMA-617 harbor at least one deleterious mutations (average of 2.2 per patient) in genes involved in DNA repair, including ATM, CHEK2, TP53, and BRCA2 [111]. The knowledge of these deleterious mutations is crucial for patient selection for TAT and for designing rational combination therapy that will exploit these genetic mutations for a better treatment outcome. Olaparib is a poly-[ADP-ribose]-polymerase 1 (PARP-1) inhibitor, a group of enzymes involved in repairing DNA breaks. Olaparib monotherapy was shown to prolong imaging-based PFS in men with mCRPC harboring alterations in at least one of BRCA1, BRCA2, or ATM genes [112]. The ability of olaparib to potentiate the antitumor effect of ^177^Lu-PSMA is currently being evaluated (NCT03874884). Immunotherapy with immune checkpoint inhibitors is a new addition to the armamentarium of cancer therapy options. Limited success has been achieved with the use of immunotherapy agents in the treatment of mCRPC. This limited success has been adduced to the immunological coldness (lack of immune cell invasion into the tumor) of prostate cancer resulting from poor expression of neoantigens [113]. Cytocidal effect of radionuclide therapy with alpha or beta-emitting radionuclide may be useful in releasing tumor neoantigen, thereby sensitizing host cellular immunity [114]. Czernin and colleagues showed, in a mouse model of mCRPC, that a combination of ^225^Ac-PSMA-617 and an inhibitor of PD-1 (program death-1) achieved better tumor control than monotherapy with either agent alone [115]. More research is needed to develop rational combinatorial therapies with synergistic effects and without overlapping toxicities for mCRPC.

## Rechallenge options

Disease progression may occur in patients who initially show satisfactory response to ^225^Ac-PSMA therapy. As therapy alternative options may be limited or already exhausted in such patients, a rechallenge with ^225^Ac-PSMA may be considered. This consideration for rechallenge should be done within the context of a multidisciplinary tumor board. For patients to be considered suitable for rechallenge, such patients must have shown demonstrable response with no prohibitive severe treatment-induced toxicities to the earlier ^225^Ac-PSMA RLT. As part of the consideration for rechallenge, a determination must be made of the cumulative dose already delivered to target organs, especially the salivary glands, bone marrow, and the kidneys, so that the maximum tolerable doses to these organs are not exceeded. This caution becomes essential, especially in patients with a good prognosis and expectation for prolonged survival.

## Where next?

Issues that are essential for the development of ^225^Ac-PSMA RLT are as follows:

- Increased production capacity by creating multiple sustainable suppliers of ^225^Ac to cater for a global demand that is likely to increase tremendously in the near future. Morgenstern and colleagues recently reviewed the current issues around supply and efforts to expand production capacity [116].
- Standardization of the techniques for GMP (good manufacturing practice) production of ^225^Ac-PSMA.
- Development and standardization of imaging techniques to allow for in vivo biodistribution studies in humans for accurate dosimetry.
- Performance of prospective multicenter studies followed by randomized control trials to validate the efficacy and safety of ^225^Ac-PSMA RLT against the current standard of care and define its place in the treatment algorithm of mCRPC. This should be with the eventual goal of securing regulatory approval for routine clinical applications.

## Conclusion

This review paper sought to describe the current global experience with the application of ^225^Ac-PSMA RLT in the treatment of mCRPC. We provided a brief background to the most promising alpha-emitting radionuclides for potential application in TAT of mCRPC and the factors militating against the clinical translation of some of them. Importantly, we presented a detailed discussion on the practicality of patient selection, therapy administration, and patient follow-up for response assessment and detection of treatment-related side effects. The currently available evidence suggests that ^225^Ac-PSMA RLT is safe and efficacious in the treatment of mCRPC, even when applied in particularly challenging clinical situations. It is suitable for salvage therapy in patients who have failed other lines of therapy, including PSMA-based RLT with ^177^Lu-PSMA. These promising results have created the much-deserved excitement for the widespread clinical application of this novel therapy and for designing rational combinatorial approaches in its application alongside other agents for more effective therapy. Achieving these visions will require integration across many disciplines, including urology, oncology, radiology, radiochemistry, and nuclear medicine. Quality prospective data accrued in trial settings will be needed to validate the efficacy of PSMA-based TAT and to situate it in the treatment sequence of mCRPC.

## Data Availability

Not applicable.
